# Association between Fruit and Vegetable Consumption and Depression Symptoms in Young People and Adults Aged 15–45: A Systematic Review of Cohort Studies

**DOI:** 10.3390/ijerph18020780

**Published:** 2021-01-18

**Authors:** Putu Novi Arfirsta Dharmayani, Melissa Juergens, Margaret Allman-Farinelli, Seema Mihrshahi

**Affiliations:** 1 Department of Health Systems and Populations, Faculty of Medicine, Health and Human Sciences, Macquarie University, Sydney, NSW 2109, Australia; putu-novi-arfirsta.dharmaya@hdr.mq.edu.au; 2 Discipline of Nutrition and Dietetics, School of Life and Environmental Sciences, Faculty of Science, University of Sydney, Sydney, NSW 2006, Australia; mjue0385@uni.sydney.edu.au (M.J.); margaret.allman-farinelli@sydney.edu.au (M.A.-F.); 3 Prevention Research Collaboration, Sydney Medical School & Sydney School of Public Health, University of Sydney, Sydney, NSW 2006, Australia

**Keywords:** fruit, vegetables, depressive symptoms, depression, young people, young adult, nutrition, diet

## Abstract

Higher consumption of fruit and vegetables has been associated with a lower risk of various chronic diseases including coronary heart disease, obesity, and certain cancers. Recently, fruit and vegetable intake has also been linked with mental health, including depression; however, this area is largely unexplored studies in young people and adults. This systematic review aimed to evaluate the association between fruit and vegetable intake and depressive symptoms in young people and adults aged 15–45. The review used a predefined protocol registered with International Prospective Register of Systematic Reviews (PROSPERO) database (ID no: CRD42018091642). The systematic review focused on peer-reviewed cohort studies published from 1 January 2000 to 31 August 2020 using searches of six electronic databases. The exposure was fruit and vegetable consumption analysed both separately and/or together, and the outcome was depression or depressive symptoms. Data from eligible studies were extracted according to predefined criteria and the studies were appraised using the Newcastle–Ottawa Scale (NOS) for cohort studies to evaluate for study quality and risk of bias. A total of 12 studies from seven countries were deemed eligible and included in the qualitative synthesis, one study was categorised as “very good” quality, nine studies were “good” quality, and two studies were “moderate” quality by the quality assessment based on the total score for the NOS. The majority of cohort studies support the evidence that fruit consumption is associated with decreased risk of developing depression. However, the inconsistent results were observed when the effects of vegetable consumption were analysed independently, and the effects of fruit and vegetables combined were analysed. Despite this, the evidence seems to be building that a possible association exists, and this may have implications for addressing the burden of mental illness in young people and adults aged 15–45 years. More well-designed prospective cohort studies are needed to provide more robust evidence on the relationship between fruit and vegetable intake and depression.

## 1. Introduction

Depression is a debilitating, chronic, and reoccurring condition that has become a major public health concern worldwide. In 2017 it was estimated that 300 million people, accounting for 4.4% of the global population, suffered from this disorder [1,2]. The number of incident cases of depression increased by almost 50% between 1990 and 2017 [3]. Major depressive disorder (MDD) is the second leading cause of years lived with disability and a large contributor to the global burden of disease globally [2], making it a substantial economic burden for countries. The peak age of onset of mental disorders, including depression, typically occurs during a period of life transition in early adulthood making it an important time for prevention strategies [4,5,6]. The percentage change in number of disability adjusted life years (DALYs) between 1990–2009 in depressive symptoms has risen dramatically in those aged 10–24 and aged 25–49, an increase of 20.7% and an increase of 53.2%, respectively [7]. Moreover, suicide is the second leading cause of death in young people aged 15–29 years in 2016 [8]. These alarming numbers emphasise the importance of preventing the onset on mental disorders as a priority for public health intervention.

The complex nature of the disease makes it difficult to attribute to a particular cause but depression has been linked to numerous biopsychosocial and lifestyle factors [9]. There has been growing interest recently in the plausible role of dietary factors as protective factors against depressive symptoms. Several systematic reviews of diet–depression studies have concluded that adhering to healthy dietary patterns is associated with reduced risk of depressive symptoms [10,11,12,13]. Particularly, higher fruit and vegetable consumption is widely recognized being important for improving mental health status [14,15,16]. However, the findings from previous studies on the association between the intake of fruit and vegetables and depression in young adults have tended to have contradictory conclusions. Some studies showed that higher intake of fruit and vegetables was associated with a lower likelihood of depressive symptoms [17,18,19], whereas other studies found no significant associations [20,21,22]. Recent systematic reviews indicated that an increase of fruits and vegetables intake may protect against the risk of depression and depressive symptoms in adults [23,24]. In older adult (>50 years) populations, many studies have shown associations between fruit and vegetable consumption and lower odds of depression development [25,26,27]. A recent systematic review in adolescents concluded that a potential of positive association between fruit and vegetable intake and mental health [28]. Contrarily, a 2016 systematic review conducted in children and young people aged 18 years and below observed that most studies found no significant association between fruit and vegetable consumption and mood [29].

The exact mechanisms by which fruits and vegetables are thought to lead to a decreased risk of depression are yet to be precisely identified. However, there are some evidence of an association with nutrients such as magnesium, zinc, and antioxidants such as vitamin C, E, and folate, found in these foods [30,31,32,33,34,35]. One possible pathway involves folate, a common vitamin found in foods such as leafy green vegetables, legumes, beans, and citrus fruits. Folate plays a critical role in the regeneration of tetrahydrobiopterin (BH4) and re-methylation of homocysteine which leads to the production of S-adenosylmethionine (SAMe) [33]. Moreover, both SAMe and BH4 are essential cofactors in the production of neurotransmitters such as serotonin, dopamine, and epinephrine, all which play a critical role in mood regulation. Several research studies exist linking folate deficiencies to depression [33,36,37,38].

Existing systematic literature reviews examining the diet–depression relationship tend to be inclusive of all study designs, and most of the included studies are cross-sectional studies [12,39,40,41,42]. This poses a methodological limitation when inferring causation in the context of diet–depression relationship [10]. Moreover, this limitation may contribute to inconsistent findings in previous systematic reviews [40]. Cohort studies have an advantage in that they are prospectively studying the associations between diet and depression and a clearer sequential relationship can be seen between exposure and outcome. Few studies have explored the relationship, especially in young people and adults aged 15-45. They remain a relatively neglected age group compared with children and older adults in diet–depression research, although the proportion of persons with depression in this age group is seen to dramatically increase [43,44] and healthy behaviour is more likely to decline during the transition to adulthood [45]. Moreover, a systematic review highlighted that emerging adulthood is a risk period for both low diet quality, including inadequate fruit and vegetable intake, and poor mental health [46]. In Australia, the 12 month prevalence of mental disorders in people aged 16–44 years was approximately 25% [47]. In this systematic review, we aimed to evaluate the association the between fruit and vegetables intake and depressive symptoms in young people and adults aged 15–45 using longitudinal cohort studies.

## 2. Materials and Methods

### 2.1. Study Design

The PRISMA framework was used to guide the reporting of methodology and outcomes [48]. The review used a predefined protocol registered with International Prospective Register of Systematic Reviews (PROSPERO) database (ID no: CRD42018091642) and can be accessed at: https://www.crd.york.ac.uk/PROSPERO/display_record.php?RecordID=91642.

### 2.2. Eligibility Criteria

The detailed inclusion and exclusion are shown in Supplementary Table S1. The following criteria was used—(1) studies in healthy individuals aged 15–45 years old. This age group was chosen because they are more likely to be a healthy population with less occurrence of chronic disease compared to mid-aged and older adult populations. During this period of life stage, key life transitions occur and their health behaviour, including diet, may change. (2) study design was restricted to original cohort studies with a follow up time of a year or longer. Due to the nature of depression, long-term follow up may well be desirable and appropriate to allow sufficient time to ascertain the occurrence of depression. Usually 12-month prevalence estimates are used to present the prevalence of mental disorders. (3) the exposure was fruit and vegetable intake analysed both separately and/or together; and (4) the outcome of studies was depression or depressive symptoms.

Studies were excluded if (1) they had study designs other than a longitudinal cohort; (2) they were published prior to 1 January 2000 as more recent studies include time-period where the prevalence of depression has increased dramatically. (3) they had follow up period <1 year; (4) major dietary patterns were examined without separate analysis of fruit and/or vegetables; (5) the studies were published in languages other than English; and if the populations included in the studies (6) had pre-existing conditions (i.e., any health problems that exist at an earlier time, including depression); (7) they had specific nutritional needs; and (8) they were unique populations which are less likely to be representative of the general population (e.g., monks).

### 2.3. Search Strategy

The systematic review focused on peer-reviewed cohort studies published from 1 January 2000 to 31 August 2020. Prespecified search terms and Medical Subject Headings (MeSH) terms were utilised to identify potentially relevant articles from six databases, namely Medline, EMBASE, PreMedline, and PsycINFO via Ovid, CINAHL via EBSCO, and Scopus. Two reviewers (PNAD and MJ) conducted a comprehensive search using the following keywords: (a) type of food (‘fruit’ OR ‘vegetable’ OR ‘FV’), (b) ‘consumption’ OR ‘intake’, (c) mental disorders (‘depression’ OR ‘depressive disorder’ OR ‘depressive symptoms’), (d) population (‘young people’ OR ‘young adults’ OR ‘adults’), and (e) study design (‘longitudinal stud*’ or ‘cohort stud*’). Full details of the search strategy can be found in Supplementary Table S2.

### 2.4. Study Selection

**Figure 1:**
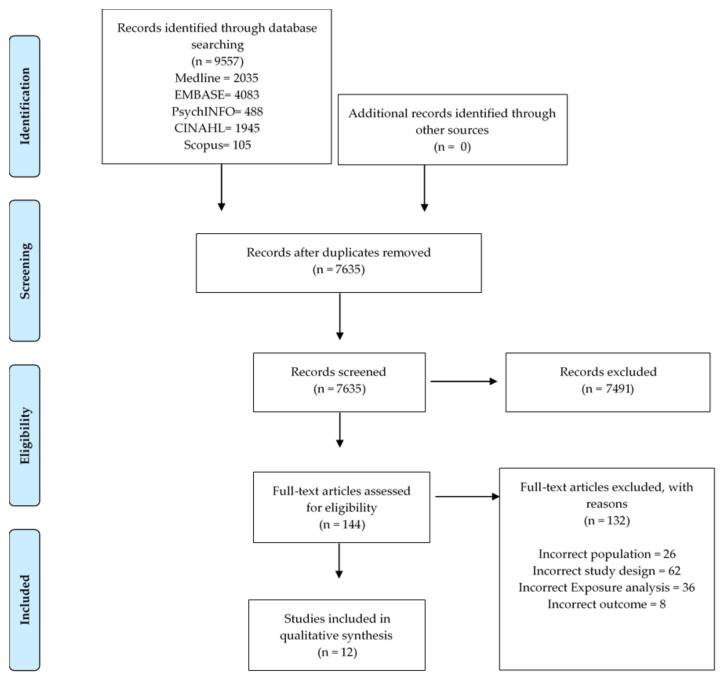
Flowchart of identification and screening process for selection of cohort studies exploring the association between fruit and vegetable intake and depressive symptoms in young people and adults aged 15–45 years.

All studies identified were imported into EndNote X9 citation management software (Thomson Reuters, Toronto, Ontario, Canada). At the first stage studies were verified and screened based on title and abstract by two reviewers (PNAD and MJ). At the second stage, all potentially relevant studies were independently screened and scrutinised for eligibility by three reviewers (PNAD, MJ, and SM). Any discrepancies were resolved by discussion between researchers. Reference lists of eligible studies were manually searched to identify any additional studies. The procedure of identification and screening process for selection of cohort studies is presented in Figure 1.

### 2.5. Data Extraction

Data extraction was independently conducted by two reviewers (PNAD and MJ) and then validated by SM using a pre-determined data extraction table. Key study components were extracted including (1) characteristics of participants (age, gender, and country of origin), (2) study details (cohort assessed, number of participants, and follow up period), (3) outcome and exposure assessment methods (diet and depression), (4) main results (including β coefficients, OR, HR, 95% CI, and *p* values), and (5) confounding factors (including all models of adjustment).

### 2.6. Quality Assessment

Two researchers (PNAD and SM) independently appraised the study quality and risk of bias of each eligible studies using the Newcastle–Ottawa Scale (NOS) for cohort studies [49]. This framework used following criteria to categorise studies: Selection (scale from 0 to 4), comparability (scale from 0 to 2), and outcome (scale from 0 to 3). The overall score of each included study was used to categorise studies as: “very good” quality (8–9 NOS points), “good” quality (6–7 NOS points), “moderate” quality (4–5 NOS points), and “low” quality (0–3 NOS points) [50]. Differences in scores regarding the quality assessment were resolved by discussion and consensus between the two researchers.

## 3. Results

### 3.1. Search Results

A total of 9557 potentially relevant studies were identified using the search strategy on six electronic databases, (Medline = 2035, EMBASE = 4083, PsycINFO = 488, PreMedline = 901, CINAHL = 1945, and Scopus = 105) which was reduced to 7635 after removal of duplicates. After abstract and title screening in the first stage, 7491 studies were excluded leaving 144 full-text articles. Of the 144 studies that were screened in full-text publications for eligibility, 12 studies were deemed eligible and included in the qualitative synthesis [51,52,53,54,55,56,57,58,59,60,61,62]. No additional articles were retrieved from reference list searching. The flowchart in Figure 1 displays the process of selection. Those full-text studies which were deemed ineligible with reasons are reported in Supplementary Table S3.

### 3.2. Study Characteristics

Table 1 shows the study characteristics. Most of the included studies were conducted in European countries; three were based in the United Kingdom (UK) [53,55,60], one from France [57], one from Spain [62], and one from Sweden [54]. Three studies were conducted in the United States of America (USA) [56,59,61], and the remaining studies were each located in Japan [51], Canada [58], and Australia [52]. The earliest publication date was 2009 and the most recent 2020. Duration of the study follow-up period ranged from 2–14 years with the number of participants varying from 139 to over 45,000. The age ranges at baseline of some of the studies also included participants that were outside the 15–45 years age group, although they were recruited in this age range, as was the nature of the longitudinal cohort. One commentary study [52] was also included as an eligible study because it presented an extra evidence regarding depression and anxiety using the same cohort data from previous study conducted by the same authors [63]. In terms of analysis, one study analysed only vegetable intake [51], and one study exclusively analysed fruit intake [54], while the rest analysed both fruit and vegetable intake [52,53,55,56,57,58,59,60,61,62]. One study consisted of only female participants [59] while the rest comprised of individuals from both genders [51,52,53,54,55,56,57,58,60,61,62]. One study [60] reported raw data from only female participants as results in the male participants were not significant. This study also separated analysis into measurements taken at two different time points: 5-years and 10-years.

### 3.3. Dietary Measures

Semi-quantitative food frequency questionnaires were used to assess diet and fruit and vegetable intake in four studies [51,59,60,62], two studies used country specific food frequency questions, one from the National Cancer Institute [61] and the other from the USA Centres for Disease Control and Prevention [58]. Multiple 24-h recalls were used in another [57], a 4d diet diary in one study [55], and self-reported questionnaires in four studies [52,53,54,56]. When looking at the analysis of exposure, three analysed the impact fruits and vegetables had independently on depression [56,60,62], five combined fruits and vegetables in their analysis [52,55,57,58,61], one looked specifically at only flavonoid containing foods which included fruits and vegetables [59], and one study analysed both the impact of fruit and vegetable together and separately [53].

### 3.4. Depression Measures

Depression assessment methods varied between studies. The most commonly used screening instruments to measure depressive symptomatology were the General Health Questionnaire (GHQ-12) [51,53,54], and the Centre for Epidemiologic Studies Depression Scale (CESD), two studies used CESD-20 [57,60], and one used both CESD-20 and CESD-10 in different waves of the study [56]. Other instruments included Composite International Diagnostic Interview-Short Form (CIDI-SF) [58], and the Moods and Feelings Questionnaire (MFQ) [55]. One study used a Short Form (SF-12) Health Survey [61], and was included as it had some measures of depression and mental health. Two studies included questions about diagnosis of depression and/or use of antidepressant medication [52,62] and another used a combination of methods across different assessment time periods [59].

### 3.5. Quality Assessment

The Newcastle–Ottawa Scale for cohort studies was used to assess the study quality. The detailed results of the quality assessment are seen in Supplementary Table S4, where the total score for the Newcastle–Ottawa Scale is given. One study was deemed as “very good” quality [58], the majority of studies (n = 9) categorised as “good” quality [51,52,53,54,55,56,59,60,62], and two studies were categorised as “moderate” quality [57,61]. Most of the studies failed to meet the requirements on ascertainment of exposure and assessment of outcome because written self-report was used to collect the exposure and outcome information, which was likely to introduce bias. Furthermore, the studies of moderate quality [57,61] only received one point each for selection and outcome criteria. No studies were excluded on the basis of their quality assessment.

### 3.6. Outcomes

A pooling of the results was not possible because the differences in the ways that analysis were conducted, and outcomes were reported and thus, results were tabulated and described qualitatively. Key results are presented in Table 2 and the summary of findings in Table 3.

#### 3.6.1. Impact of Fruit and Vegetable Intake on Depressive Symptoms

Two good quality studies and one moderate quality study explored the impact of fruit and vegetable intake on depressive symptoms [54,56,59]. Two studies used the CES-D instrument [57,60], and one study used the MFQ to assess depressive symptoms [55]. At an individual study level, in the 5-year analysis of the Whitehall II cohort [60], a greater consumption of both vegetables and fruit decreased the odds of recurrent depressive symptoms in women while no significant results were seen in men. In the 10-year analysis an improvement or maintenance of vegetable intake was associated with reduced odds of recurrent depressive symptoms in women, while women whose consumption decreased over the same time frame had higher odds of recurrent depressive symptoms. Similarly, an increased fruit intake decreased the odds of depression development in the 10-year analysis, however, no significant association was observed when women maintained or decreased fruit intake. In contrast, Winpenny et al. [55] reported no prospective association between fruit and vegetable intake at age 14 years and depression symptomatology at age 17 years after adjusting for risk factors, such as depressive symptoms at baseline, socio-economic status, physical activity, and total energy intake. Similarly, the finding from the Supplementation en Vitamines et Mineraux Antioxydants (SU.VI.MAX) study [57] illustrated that adherence to the French nutrition guidelines resulted in a decrease in depressive symptom development due to an overall healthy diet. This was ascertained by comparing the guidelines score without inclusion of fruit and vegetables and the result was still protective.

Four studies investigated the impact of fruit and/or vegetable intake independently on more general measures of depressive symptoms including mental health measures [51,53,54,61]. Three good quality studies used the GHQ-12 [51,53,54] and one moderate study used the SF-12 Health Survey [61]. In the Japan Multi-Institutional Collaborative Cohort (J-MICC) study [51], an inverse association between vegetable intake and a GHQ score ≥ four was not significant after controlling for confounders. In contrast to that, the study from Sweden highlighted that daily consumption of fruit and berries was a substantial determinant to predict stability in mental health among the 18–29 age group [54]. Similar results were observed in a longitudinal UK study [53] which showed the importance of both frequency and quantity of intake of fruit and vegetables on good mental health. It was highlighted that daily consumption of fruit and/or vegetable (at least one portion) contributed to maintenance of good mental health. Moreover, frequent vegetable consumption had a more substantial effect on mental health than frequent consumption of fruit. One moderate quality study that assessed emotional and mental health found that fruit and vegetable consumption had no significant association with mental health, and the results indicated that increased physical activity had a positive association with better mental health, irrespective of fruit and vegetable consumption [61].

#### 3.6.2. Impact of Fruit and Vegetable Intake on Depression

One very good quality study and four good quality studies examined the association between fruit and vegetable intake and depression, of which four studies measured the depression doctors diagnosis or medication use [52,58,59,62], and one study used the CES-D instrument [56]. Two good quality studies reported the protective effect of fruit intake against risk of developing depression [59,62]. Moreover, a significant protective effect was observed from greater intakes of legumes and “fruits and nuts” [62]. Citrus fruits and juices were also shown to reduce the odds of depression in women from the Nurses’ Health Study [59]. However, a greater intake of vegetables had no significant effect on the development of depression in individuals in the Seguimiento Universidad de Navarra (SUN) study [62]. The very good quality study showed fruit and vegetable intake not to be associated with depression development or distress after adjusting for the key confounders [58]. Contrastingly, Mujcic and Oswald [52] found an inverse association between fruit and vegetable consumption and the probability of being diagnosed with depression/anxiety within the next 24 months. An interesting finding in the Add Health cohort [56] was that the association between fruit consumption during adolescence and reduced odds of depression in adulthood was not significant after adjustment for adolescent depression. Contrary to that, consuming vegetables once a day was significantly related to reduced odds of adult depression among females after adjustment for adolescent depression, but not at higher intakes (twice or more a day). However, this association was substantially attenuated after further controlling for relevant confounders.

## 4. Discussion

This systematic review aimed to evaluate the association between fruit and vegetable intake and depressive symptoms in young people and adults aged 15–45 years. We identified 12 cohort studies from seven countries. Among six cohort studies which analysed the impact of combined fruit and vegetable consumption [52,53,55,57,58,61], only two studies demonstrated an inverse association with depressive symptoms [52,53]. Varying results were observed when the effects of fruit and vegetables were analysed separately. Among the five good quality studies which showed an inverse association between fruit consumption and depression after adjusting for key confounders [53,54,59,60,62], two good quality studies showed that the association was observed in women [59,60]. Two good quality studies showed an association between vegetable consumption and lower risk of development depression [53,60]. Two good quality studies showed no association between vegetable consumption and depression [51,62]. One very good quality study and one good quality study, although initially showing a significant association in unadjusted models, showed no association between fruit and vegetable intake and depression after adjustment for confounders, such as obesity, physical activity, and BMI [56,58]. These results suggest that fruit and vegetable independently may lead to different effects on mental health. Two moderate quality studies showed no association between fruit and vegetable intake and depression symptoms [57,61]. Two good quality studies reported possible gender effects, because there were different impacts of fruit and vegetable consumption on depression between women and men [56,60]. Although we are unable to make definite assertions, the evidence seems to be building that a possible association exists, and this may have implications for addressing the burden of mental illness in young people and adults aged 15–45 years.

A common finding across the 12 studies was the low intake of fruit and vegetables generally, and the failure to meet recommended guidelines. According to the Understanding Society study [53], 87% of 15–29 year-olds and 80% of 30–41 year-olds consumed fewer than five portions a day in the UK. Despite the low rate of adherence to guidelines, the study found that even small increases in the consumption patterns of individuals may translate into substantive positive effects for the well-being [53]. Furthermore, the more often fruits and vegetables are eaten in a week, the better mental well-being is likely to be. The study by Winzer et al. [54] for example concluded that consuming fruit and berries on daily basis is a determinant of stability in mental health in the 18–29 age group. Another study also illustrated that vegetable consumption once a day among women was prospectively associated with a reduced risk of adult depression although the association diminished after further adjustment for key confounders [56]. These findings indicate that even one portion of fruit and vegetable daily is likely to reduce the risk of depression in adults. It is notable that the frequency with which fruit and vegetables are consumed is crucial. An additional finding was that depression did not predict lower consumption of fruits and vegetables longitudinally [52,58].

The most salient findings were that the independent effects of fruits and vegetables on depression and depressive symptoms differed when analysed separately. The inverse relationship was more likely to be observed between fruit intake and depressive symptoms than vegetable intake. Such disparities were also seen in a study conducted in Australian middle-aged women [65]. A higher intake of fruits was seen to be protective of depressive symptoms in both cross sectional and longitudinal analyses whereas vegetables were only protective using cross sectional analyses. Authors from this study suggested that the discrepancy between the two food groups could be a result of differing chemical composition, whereby fruits tended to contain higher levels of antioxidants and anti-inflammatory components such as carotenoids, flavonoids, and resveratrol. While this is a possibility, it may also be hypothesised that in these studies the vegetable intake is not consumed in quantities high enough to see protective effects, or that the types of vegetables consumed in larger amounts do not contain the corresponding nutrients suggested to be protective. Therefore, further studies are required to observe the independent effects of fruit and vegetables on depression and depressive symptoms.

Interestingly, in two good quality studies, there was evidence of differences in the association according to gender [56,60]. The results demonstrated that vegetable consumption among females is inversely associated with future depressive symptoms. The association was attenuated in a US study by further adjustment for potential confounders [56], while the other remained significant [60]. In terms of fruit consumption, no significant association was observed in both genders in the US study [56], whereas an inverse association was significantly observed in women only in the Whitehall II study from the UK [60]. A plausible explanation for the gender differences, may be explained by psychosocial factors. According to Emanuel et al. [66], a gender difference in fruit and vegetable intake may be attributable to attitudes and perceived behavioural control. Women reported more favourable attitudes and perceived behavioural control towards fruit and vegetable intake than men.

While all studies adjusted for most key potential confounders, it is difficult to say with certainty that all confounding factors were accounted for, due to the complex nature of both depression and diet. For instance, the studies that showed protective effects against depression did not adjust for BMI in their analysis [53,54,60]. BMI has been shown to have a non-linear association with depression, whereby those who are underweight or obese tend to have higher odds of depression compared to individuals that are normal weight or overweight [67,68]. Thus, failure to adjust for BMI, despite adjustment for central obesity, may have obscured the effects observed in the Canadian longitudinal study [58]. Additionally, none of the included studies adjusted for fish intake in their analysis. Greater intakes of fish and omega-3 fatty acids have been associated with lower odds of depression development and thus could be an important confounding factor [69,70,71]. Not adjusting for all confounders may mean that the associations observed are a result of an unmeasured variable and thus not a true reflection of the association between exposure and outcome. This influence was evident in two included studies [56,58] where consuming vegetable once a day were shown to be protective against depression development in women until adjustments of age, household income, ethnicity, physical activity, and body mass index were made [56], and fruits and vegetables were shown to be protective against depression development until additional adjustments of social support, obesity, and smoking were made in Canadian longitudinal study [58].

Different measurement tools in exposure and outcome may also contribute to the current conflicting results. This may have contributed to the variations in results seen. There were large variations in the methods used to assess intake of fruits and vegetables. For example, four studies used serving size [55,59,60,61], while three studies used frequency of consumption [53,54,58], and another used meeting the national guidelines [51]. A study by Offringa et al. [72] illustrated that the total fibre content of vegetables consumed by 100-kcal portions was significantly higher compared to the total fibre content of vegetables consumed per serving. Therefore, this difference may interfere with the impact of vegetable consumption on predictive depressive symptoms. The dynamic and varying nature of diet means that there may be unavoidable errors in dietary measurements. Therefore, it is important to determine the strengths and weaknesses of the assessment methods used within each study. Reporting bias may be present in all studies as food intake relies on the participants’ recall ability and assumption that reported intake close to the true intake. Participants may be inclined to over emphasize their usual intake towards including healthier food choices as this appears more socially desirable [73]. Often, the use of a sensitivity analysis would tend to negate the effects of misreporting, however, none were conducted in any of the included studies. Validation of the dietary assessment method is therefore necessary to ensure the results are accurate. Despite the individual dietary assessment methods having their own limitations, most of the included studies used validated tools. Similarly, different instruments across the studies were used for defining depression, which not limited to clinical diagnosis or antidepressant use.

In spite of the use of validated instruments, such as the CES-D and GHQ-12, using different ways of scoring and cut-off points might affect the results [4]. According to Fried [74], lack of content overlap among common depression scales may pose a threat to the generalisability and replicability of depression research. The results of depressive symptoms assessed with the CES-D are less likely to generalise to other depression scales due to idiosyncratic items and lack of overlap [74]. Each depression scale was developed for different purposes, for instance, the CES-D was developed specially to screen for depression in a general population setting [75]. Similarly, the GHQ-12 was widely used in the general population and non-psychiatric settings, however in terms of screening, it is not limited to identify depression, but also common mental disorders [76]. Significant results were observed when the screening of depression was using both self-reported depression scale and clinical diagnostic (doctor diagnosis or antidepressant use) [52,59,60,62]. Possibly, the differences in content between self-reported depression scale and clinician assessment may lead to lower the agreement on diagnosis of depression, which affect the results [77,78,79]. Therefore, the utilization of clinician assessment and self-reported depression scale in future studies is recommended to achieve the most accurate prediction of depression.

Existing literature reviews conducted on the diet–depression relationship have shown varying results. While some have illustrated positive effects around healthy dietary patterns and decreased risk of depression [10,12,24], others have detected no association [41]. Furthermore, in line with the results obtained from this review, most existing work recommended the need for additional research in this area, especially studies with cohort designs [40,42,80]. To address one of the methodological limitations in previous systematic reviews, the eligible studies were only those studies conducted with a longitudinal cohort design, with the hope of obtaining a clearer sequential relationship and stronger causal inferences between fruit and vegetable consumption and depression symptoms. However, a concrete conclusion is unable to be drawn. The lengths of follow-up duration varied across the studies but overall, no difference effects were found. It is nevertheless noteworthy that the unadjusted potential confounders and different measurement tools probably have stronger effects on contradictory results.

There have been few randomised controlled trials (RCT) which have assessed the relationship between fruit and vegetable intake and depression. Although experimental evidence is the best study design to assess causal effects, most RCTs are very short term and are performed in populations with existing illness. For example, an intervention study to increase fruit and vegetable consumption in young adults with low fruit and vegetable consumption indicated that fruit and vegetable consumption improved several aspects of psychological well-being [16] but this was only carried out over 14 days. Two RCTs showed significant reductions in depressive symptoms in young adults with depression symptoms after a brief diet intervention [81,82]. However, the diet intervention was designed to comply with healthy diet recommendations, and fruit and vegetables were consumed along with other food groups. Additionally, the target populations were young adults with depression, which differed with the healthy population in our systematic review.

This study looks solely at the impact of fruits and vegetables because few systematic reviews [42] examined its impact on depression and most of them have examined the impact of dietary patterns on depression. We chose this option because findings will be able to be translated more readily into recommendations for whole foods. In addition, this review exclusively examined the association in young people and adults aged 15–45 years which is the age transition where most mental health issues arise.

### Strengths and Limitations

The exclusive use of cohort studies poses both a limitation and strength to this review. The inclusion of only a single study design is a form of selection bias. However, by reviewing only cohort studies, the direction of causation is easier to infer as there is more likely to be a temporal association between exposure and outcome, provided subjects with the outcome at baseline are excluded from the analysis. Additional strengths of this review include the comprehensive nature of the search strategy, thorough quality analysis and the large sample sizes seen in 10 of the 12 studies may have led to a higher statistical power to ascertain an effect. Importantly, this systematic review provides results of the independent effects of fruit and vegetable separately enabling us to recognize any differential effects which adds to the research base on this topic. To our knowledge, this is the first systematic review of cohort studies to evaluate the association between fruit and vegetable intake and depressive symptoms in young people and adults aged 15–45.

The strict inclusion criteria for our systematic review resulted in only 12 studies being eligible for review and between studies, there were also considerable methodological differences which made direct comparisons challenging. Most of included studies used dietary recall to assess fruit and vegetable consumption. This methodology might be liable to recall bias although it has higher precision in assessing dietary intakes. In terms of exposure assessment, measurement of fruit and/or vegetable consumption varies from grams/day, to portions, serving sizes, quantity, and frequency. Different instruments were used across studies to assess depressive symptomatology, which not limited to clinical diagnosis or antidepressant use. Inconsistency in the outcome measures resulted in different definitions of depression or depressive symptomatology, which also might affect the results. Further, the validity of results may be limited because the age range in majority of studies were slightly extended outside of young people and adults aged 15–45 years. Other limitations include language bias, as studies published in languages other than English were excluded and publication bias may be present.

## 5. Conclusions

Despite a paucity of cohort studies, this study complements existing work evaluating the evidence on the impact of fruit and vegetable consumption on depression. Unique aspects of this systematic review include the exclusive use of cohort studies, specifically in young people and adults aged 15–45. Our findings highlight the potential importance of the association between fruit and vegetable consumption and depressive symptoms in young people and adults aged 15–45 years. There is inconclusive evidence on the effect fruits and vegetables have on reducing the odds of developing depression and depressive symptoms. More robust evidence is needed to address the specific aspects of diet that could prevent the development of depression. Thus, we recommend the diet–depression relationship be examined in well-designed prospective cohorts as well as randomised controlled trials. With the evidence building there is potential to inform public policy and add positive mental health outcomes to an already extensive list of reasons as to why people should prioritise a healthy diet.

## Figures and Tables

**Table 1:** Characteristics of included studies.

Reference/Country/Year	Participant Characteristics (Age Range, Mean Age at Baseline (SD), Gender)	Study Characteristics (Number of Participants, Follow-Up Period)	Cohort	Dietary Assessment Method (Recall Period)	Depression Assessment Method (Analysis Assessment)
Choda et al. [51] Japan, 2020	Age range at baseline: 35–69 y Age mean: Participants with a GHQ score ≥ 4 is 50.1 (9.1); participants with a GHQ score < 4 is 52.9 (9.5) Gender (female): ~50%	4701 ~5 y (Daiko) ~6 y (Shizuoka)	The Japan Multi-Institutional Collaborative Cohort (J-MICC), the Daiko Study and the Shizuoka area	A validated short FFQ (46 food items) (over the past years)	The 12-item General Health Questionnaire (the GHQ-12) (psychological distress and social dysfunction factors)
Mujcic and Oswald [52] Australia, 2019	Age range: ≥15 y Age mean:-^a^ Gender (female):-^a^	7108 2 y	The Household, Income, and Labour Dynamics in Australia (HILDA) Survey	*Short questions on usual intake and frequency intake*: “how many days in a usual week do you eat fruit?” and “how many days in a usual week do you eat vegetables?” “On a day when you eat fruit, how many serves of fruit do you usually eat?” and “On a day when you eat vegetables, how many serves of vegetables do you usually eat?” [63]	“Have you ever been told by a doctor or nurse that you have any of long-term health conditions listed below? Please only include those conditions that have lasted or are likely to last for six months or more: Depression/Anxiety”
Ocean et al. [53] UK, 2019	Age range at baseline: 15–104 y Age mean: 47.1 Gender (female):-^a^	Over 45,000 6 y	The UK Household Longitudinal Study (UKHLS)	*Short questions on portion intake*: “on a day when you eat fruit or vegetables, how many portions of fruit and vegetables in total do you usually eat?”	The 12-item General Health Questionnaire (the GHQ-12) (psychological distress and social dysfunction factors)
Winzer et al. [54] Sweden, 2018	Age range: 18–29 y Age mean:-^a^ Gender (female): 61.8%	1704 ~12 y	The Stockholm Public Health Cohort (SPHC)	*Short questions on frequency intake*: How often do you eat Fruit and berries (an apple, an orange, a banana, a glass of juice, grapes, strawberries)?	The 12-item General Health Questionnaire (the GHQ-12) (psychological distress and social dysfunction factors)
Winpenny et al. [55] UK, 2018	Age range at baseline: 14 y Age mean: 14.5 (0.3) Gender (female): 60%	603 3 y	The ROOTS study	A 4d diet diary (two weekdays and two weekend days)	The Moods and Feelings Questionnaire (MFQ) (depressive symptoms)
Hoare et al. [56] USA, 2018	Age range at baseline: 12–18 y Age mean at baseline: 15.9 (1.7) Age mean at follow up: 28.9 (1.7) Gender (female):-^a^	3696 ~14 y	Add Health	*Short questions on frequency intake*: “How often did you eat fruit or drink fruit juice yesterday?” The same item with response options was asked for vegetable consumption (previous day)	The Centre for Epidemiologic Studies Depression Scale CES-D 20 (Wave 1) CES-D 10 (Wave 4) (depressive symptoms)
Collin et al. [57] France, 2016	Age range: 35–60 y Age mean: 49.5 (6.2) Gender (female): 56.2%	3328 11 y *	Supplementation en Vitamines et Mineraux AntioXydants (SU.VI.MAX)	Multiple (3–6) 24-h recalls (Usual intake)	CES-D 20 (Chronic or recurrent depressive symptoms)
Kingsbury et al. [58] Canada, 2016	Age range: 18–104 y Age mean: 44.16 (18.41) Gender (female): 52.8%	8353 Continuous every 2 y (1994/1995–2010/2011)	Canadian Longitudinal Survey	*Food frequency questions*: From the fruit and vegetable module in the behavioural risk factor surveillance system of the USA Centres for Disease Control and prevention (Usual intake)	CIDI-SF (Major depression) K6 (Distress)
Chang et al. [59] USA, 2016	Age ** range: 36–55 y Age mean: ~46.3 (4.6) Gender (female): 100%	36,658 10 y	Nurses’ Health Study II ** (NHSII)	Semi-quantitative FFQ (130 food items) (Previous years usual intake)	MHI-5 (1993, 1997) Antidepressant use (1997) Doctor diagnosis (2001) (Depression)
Akbaraly et al. [60] UK, 2013	Age range at baseline: 35–55 y Age at initial measurement for this analysis: 39–64 y Gender (female): 25.1%	4215 (5 y) 4053 (10 y)	Whitehall II study, UK civil servants	Semi-quantitative FFQ (127 food items) (Previous years usual intake)	CES-D 20 or/and use of antidepressant medication (Depressive symptoms)
Chai et al. [61] USA, 2010	Age range: >18 y Age mean: 55.3 (15.5) Gender (female): 74.1%	139 2 y	Multiethnic sample of adults living in Hawaii	Food Frequency questions: National Cancer Institute fruit and vegetable questionnaire. (Previous months intake)	SF-12 Health Survey (SF-12)
Sanchez-Villegas et al. [62] Spain, 2009	Age range at baseline: 18–104 y Age mean male: 42.7 (13.3)[64] Age mean female: 35.1 (10.7) [64] Gender (female): ~58.4%	10,094 4.4 y	The Seguimiento Universidad de Navarra’ Study cohort, alumni of the University in Spain (SUN cohort)	Semi-quantitative FFQ (136 food items) (Usual intake)	Positive response to “Have you ever been diagnosed as having depression by a medical doctor” or/and who reported the habitual use of antidepressant drugs. (Clinical depression)

Abbreviations: SD; standard deviation, y; years, UK; United Kingdom, FFQ; food frequency questionnaire, CES-D; Centre for Epidemiologic Studies Depression Scale, SU.VI.MAX; Supplementation en Vitamines et Mineraux AntioXydants, USA; United States of America, CIDI-SF; Composite International Diagnostic interview-Short Form, K6; Kessler psychological Distress Scale, SUN; Seguimiento Universidad de Navarra/University, NHS; Nurses’ Health Study, 4d; 4day, MHI-5; 5-item mental health index, and QOL; quality of life. * Follow up 1996–1997 to 2007–2009. ** Study included data from NHS as well however characteristics were described independently and thus those from the NHSII are only presented in this table. ^a^ This information was not provided in the publication.

**Table 2:** Description of exposure, outcome and key results of studies.

Ref	Exposure	Outcome	Key Results		
			Fruit and Vegetable	Fruit	Vegetable
[51]	Vegetable intake (frequency)	Mental Health (GHQ-12)	N/A	N/A	Model 2 *p*-trend = 0.291 Ref: Lowest quartile of exposure (Q1) OR: Q2 = 1.20 (0.95–1.50) OR: Q3 = 0.98 (0.77–1.25) OR: Q4 = 1.21 (0.95–1.55)
[52]	Fruit and vegetable (portions/day)	Depression/Anxiety (Doctor diagnosis)	β = −0.0041 (−0.008, −0.001) *p* = 0.017 **	N/A	N/A
	Reverse causality Diagnosed with depression/anxiety	Fruit and vegetable	β = −0.0718 (−0.174, 0.031) *p* = 0.170	N/A	N/A
[53]	Fruit and vegetable (portions/day) Days each week eat fruit (frequency) Days each week eat vegetables (frequency)	Well-being (GHQ-12)	Specification (3) = 0.133 *** (0.0245)	Specification (4) Ref: Never 1–3 days = 0.259 *** (0.0896) 4–6 days = 0.423 *** (0.0989) Every day = 0.613 *** (0.0982)	Specification (4) Ref: Never 1–3 days = 0.518 *** (0.171) 4–6 days = 0.803 *** (0.175) Every day = 0.925 *** (0.177)
[54]	Consumption of fruit and berries (frequency)	Mental Health (GHQ-12)	N/A	N/A	*p*-trend = 0.071 Ref: Rare consumption of fruit and berries OR: Daily consumption = 1.39 (1.05–1.84) ** OR: Weekly consumption = 1.25 (0.94–1.67)
[55]	Fruit and vegetables (servings/day)	Depressive symptoms (MFQ)	Model 3: β = 0.14 (−0.15, 0.43) Model 3 (male): β = 0.06 (−0.32, 0.44) Model 3 (female): β = 0.21 (−0.22, 0.64)	N/A	N/A
[56]	Fruit consumption (quantity/day) Vegetable consumption (quantity/day)	Depression (CES-D 10 and CES-D 20)	N/A	Model 3 (pro) In males results Ref: No fruit consumption OR: Once = 0.72 (0.46, 1.11) OR: Twice + = 0.71 (0.47, 1.07) In females results Ref: No fruit consumption OR: Once = 0.92 (0.63, 1.33) OR: Twice + = 0.73 (0.62, 1.26)	Model 3 (pro) In males results Ref: No vegetable consumption OR: Once = 1.07 (0.72, 1.57) OR: Twice + = 1.02 (0.66, 1.56) In females results Ref: No vegetable consumption OR: Once = 0.74 (0.54, 1.02) OR: Twice + = 0.80 (0.57, 1.12)
[57]	PNNS-GS ^a^ without Fruits and vegetables	Depressive symptoms (CES-D 20)	Excluding fruits and vegetables OR: 0.84 (0.77, 0.91) *** p = <0.0001 When adjusted for fruit and vegetable intake the PNNS-GS score remained statistically significant	N/A	N/A
[58]	Fruit and vegetable intake (daily frequency) Fruit and vegetable intake (daily frequency)	Depression (CIDI-SF) Distress (K6)	Model 2 (dep): β = −0.03 (−0.05 to −0.01) Model 3 (dep): β = 0.001 (−0.03 to 0.04) Model 2 (dis): β = −0.03 (−0.05 to −0.01) Model 3 (dis): β = 0.02 (−0.01 to 0.04)	N/A	N/A
[58]	Inverse association Depression Distress	Fruit and vegetable intake Fruit and vegetable intake	Model 2: β = −0.27 (−0.42 to −0.11) Model 3: β = −0.10 (−0.22 to 0.02) Model 2: β = −0.02 (−0.03 to −0.01) Model 3: β = 0.01 (−0.01 to 0.02)	N/A	N/A
[59]	Citrus fruit and Juice combined (servings/day) Citrus fruit (servings/day) Onions (servings/day)	Depression (MHI−5, antidepressant use, doctor diagnosis)	N/A	Citrus fruit and Juice combined *p* = <0.0001 *** Ref: Lowest quintile of exposure (Q1) HR: Q2 = 0.94 (0.85, 1.05) HR: Q3 = 0.89 (0.78, 1.02) HR: Q4 = 0.85 (0.75, 0.97) HR: Q5 = 0.82 (0.74, 0.91) Citrus fruit *p* = 0.001 *** Ref: Lowest quintile of exposure (Q1) HR: Q2 = 0.93 (0.88, 0.99) HR: Q3 = 0.91 (0.86, 0.96) HR: Q4 = 0.97 (0.83, 1.13) HR: Q5 = 0.87 (0.75, 1.01)	Onions *p* = 0.25 Ref: Lowest quintile of exposure (Q1) HR: Q2 = 1.00 (0.94, 1.06) HR: Q3 = 0.98 (0.92, 1.05) HR: Q4 = 0.96 (0.89, 1.02) HR: Q5 = 0.99 (0.89, 1.09)
[60]	5−year analysis (Phase 7) Vegetable intake (servings/day) Fruit intake (servings/day)	Depressive symptoms (CES−D 20 or/and use of antidepressant medication)	N/A	OR: ~0.68 ^b^ (0.58, 0.97) (Model 3)	OR: ~0.65 ^b^ (0.51, 0.88) (Model 3)
[60]	10-year analysis (Phase 9) Vegetable intake (servings/day) Fruit intake (servings/day)	Depressive symptoms (CES-D 20 or/and use of antidepressant medication)	N/A	Women’s results Ref: Those who maintained low OR: Maintaining = ~0.60 ^b^ (0.40, 1.01) OR: improving = ~0.30 ^b^ (0.25, 0.60) Ref: Those who maintained high OR: Decreasing = ~1.55 ^b^ (0.90, 3.01) (NS)	Women’s results Ref: Those who maintained low OR: Maintaining = ~0.50 ^b^ (0.30, 0.90) OR: Improving2 = ~0.45 ^b^ (0.27, 0.91) Ref: Those who maintained high OR: Decreasing = ~2.6 ^b^ (1.35, 5.35)
[61]	Fruit and vegetable intake (servings/day)	Quality of Life (SF-12)	MCS scores *p* > 0.05 at all-time points (T1–T7) Correlation co-efficient ranged from: −0.06 to 0.13	N/A	N/A
[62]	Fruits and nuts (grams/day) Vegetables (grams/day)	Depression (doctor diagnosis or antidepressant use)	N/A	*p* trend = 0.007 *** Ref: Lowest quintile of exposure (Q1) HR: Q2 = 0.69 (0.53–0.91) HR: Q3 = 0.67 (0.51–0.88) HR: Q4 = 0.69 (0.52–0.91) HR: Q5 = 0.61 (0.45–0.82) Merged Q3–Q5: 0.67 (0.54–0.84)	P trend = 0.81 Ref: Lowest quintile of exposure (Q1)HR: Q2 = 0.88 (0.67–1.17) HR: Q3 = 0.87 (0.66–1.16) HR: Q4 = 0.94 (0.71–1.25) HR: Q5 = 0.93 (0.69–1.24)

Abbreviations: N/A; not available, AHEI; Alternate healthy eating index, SD; standard deviation, OR; odds ratio, Ref; reference, NS; not significant, PNNS-GS; French Programme National Nutrition Sante’-Guidelines Score, Q; quartile/quintile, Dep; depression, Dis; distress, HR; hazard ratio, MDP; Mediterranean dietary pattern, Pro; prospective, MCS; Mental component summary score, CES-D: Centre for Epidemiologic Studies Depression Scale, GHQ; General Health Questionnaire, CIDI-SF; Composite International Diagnostic interview- Short form, K6; Kessler psychological Distress Scale, MHI-5; 5-item Mental health index, SF-12; The 12-item Short Form Survey, and MFQ; Mood and Feelings Questionnaire. ** *p* < 0.05. *** *p* < 0.01.^a^ Refer to Appendix A. ^b^ Raw data not reported- only graphically represented estimate provided.

**Table 3:** Summary of the study findings accompanied by the quality assessment based on the total score for the Newcastle–Ottawa Scale.

Ref	Summary	Quality
[51]	Vegetable consumption was not associated with GHQ score before or after adjustment of confounders using prospective logistic regression. Adequate consumption of certain nutrients and foods may lead to better mental health in Japanese adults.	Good
[52]	Individuals who increased fruit and vegetable intake from 0 to 8 portions/day were on average 3.2% points less likely to experience depression or anxiety within the next 24 months. Fruit and vegetable consumption may help to protect against future risk of clinical depression and anxiety. There is no decisive evidence on whether the current rate of depression or anxiety predicts higher or lower fruit and vegetable consumption in the future.	Good
[53]	Mental well-being responds in a dose-response fashion to increases in both the quantity and the frequency of fruits and vegetables consumed. Increasing one’s consumption of fruit and vegetables by one portion (on a day where at least one portion is consumed) leads to a 0.133-unit increase in mental well-being (*p* < 0.01). The more often fruit and vegetables are consumed in a week, the more likely individuals have a higher mental well-being.	Good
[54]	Consuming fruit and berries on a daily basis were considered as a determinant for stable mental health. Having healthy food intake, demonstrated by consuming fruit and berries, was one of six determinants to predict stability in mental health.	Good
[55]	Fruit and vegetable consumption at age 14 years were not significantly associated with depressive symptoms at age 17 years based on based on prospective logistic regression. Diet quality was not significantly associated with depressive symptoms.	Moderate
[56]	Fruit and vegetable consumption could be a protective factor against adult depression. Fruit consumption among males and vegetable consumption among females were prospectively associated with a reduced risk of adult depression in unadjusted models (Model 1). The association between vegetable consumption and reduced risk of adult depression among females remained significant after adjusted for adolescent depression, but not in fully adjusted model (Model 3).	Good
[57]	Higher adherence to the French nutritional guideline assessed by PNNS-GS ^a^ was associated with a lower likelihood of chronic or recurrent depressive symptoms. This association was not driven by any specific component of the PNNS-GS (including Fruit and vegetables) but was a result of an overall healthy diet.	Moderate
[58]	A greater fruit and vegetable consumption at the initial measurement was associated with a lower risk of depression at the next. This association was evident in Model 2 adjustments however disappeared once obesity was included in the adjustment (Model 3). Similarly, a greater fruit and vegetable consumption at the initial measurement was associated with lower distress scores at the next; however, disappeared once social support smoking and physical activity were added to the model (Model 3). Inverse association showed that depression and distress at initial measurements predicted lower consumption of fruits and vegetables at the next measurement. These associations were no longer evident once social support, smoking and physical activity were added to the model.	Very Good
[59]	Citrus intake (fruit and juice) ≥2 servings/d was associated with an 18% reduction in depression risk. ^b^ Independently both citrus fruit and juice showed significant associations to a reduction in depression. This was true for moderate to high intakes of citrus fruit but only high intakes of juice. Onion intake was not associated with depression risk. Diet higher in flavonoids results in a moderate reduction in risk of depression- especially in older women.	Good
[60]	High consumption of fruits and vegetables was associated with lower odds of recurrent depression. Improvement in fruit and vegetable score led to lower odds of subsequent depressive symptoms compared to those who maintained low scores. Decrease in AHEI ^a^ score led to higher odds of depression in vegetables but not fruit. Improvement in fruit and vegetable score led to lower odds of subsequent depressive symptoms compared to those who maintained low scores. Decrease in AHEI score led to higher odds of depression in vegetables but not fruit.	Good
[61]	There was no significant association between MCS score and daily fruit and vegetable consumption. Increasing weekly physical activity levels was significantly associated with increasing MCS at all time points. Physical activity predictive of positive mental health irrespective of other behaviours such as fruit and vegetable intake and TV/video watching	Moderate
[62]	Greater adherence to the Mediterranean dietary pattern ^a^ resulted in more than a 30% reduction in depression development. Compared to participants with the lowest consumption fruit and nut those with the highest intake had a 39% decreased risk of developing depression. There was no significant association between vegetable intake and depression. Compared to participants with lowest consumption of legumes those with highest intake had a 27% decreased risk of developing depression.	Good

Abbreviations: AHEI; Alternate healthy eating index, PNNS-GS; French Programme National Nutrition Sante’-Guidelines Score, MCS; Mental component summary score, GHQ; General Health Questionnaire. ^a^ Refer to Appendix A. ^b^ This trend was consistent for both NHS and NHSII cohort.

## Data Availability

Data sharing not applicable.

## Supplementary Materials

The following are available online at https://www.mdpi.com/1660-4601/18/2/780/s1, Table S1: Inclusion and exclusion criteria for study designs; Table S2: Full electronic search strategy applied for Medline, Embase, and PsycInfo; Table S3: Justification of exclusion at full text screening; Table S4: The Newcastle–Ottawa Scale results.

## Appendix A

### Diet Types Explained

*Alternate healthy eating index (AHEI)*: Alternate healthy eating index comprises of summing 9 component scores (fruit, vegetable, ratio of white meat (seafood and poultry) and red meat, trans fat, ratio of PUFA to SFA, total fibre, nuts and soy, alcohol consumption and long-term multivitamin use) where by a higher score corresponds to a healthier diet [60].

*PNNS-GS*: Based on the Program National Nutrition Sante’ the PNNS-GS contains 13 dietary components. 8 of the components referred to meeting the food serving recommendations, while four of the components are markers of limited consumption for recommendations without providing quantified frequency. The final components helps estimate adherence to physical activity recommendations on a daily basis [83].

*Mediterranean diet pattern (MDP)*: consists of 9 components: high ration of MUFA to SFA, moderate alcohol intake, high intake of legumes, high intake of cereal, high intake of fruit and nuts, high intake of vegetables, low intake of meat and meat products, moderate intake of milk and dairy products and high intake of fish. The score was positive for intake above the median for 6 components (MUFA/SFA ratio, legumes, cereal, fruit and nuts, vegetables or fish), and intake below the median for 2 components (meat and dairy). For alcohol, points were given if intake was within a sex specific range [62].

## References

[1] World Health Organization Depression and Other Common Mental Disorders: Global Health Estimates. http://apps.who.int/iris/bitstream/10665/254610/1/WHO-MSD-MER-2017.2-eng.pd?ua=1.

[2] Ferrari A.J., Charlson F.J., Norman R.E., Patten S.B., Freedman G.D., Murray C.J., Vos T., Whiteford H.A. (2013). Burden of Depressive Disorders by Country, Sex, Age, and Year: Findings from the Global Burden of Disease Study. PLoS Med..

[3] Liu Q., He H., Yang J., Feng X., Zhao F., Lyu J. (2020). Changes in the global burden of depression from 1990 to 2017: Findings from the Global Burden of Disease study. J. Psychiatr. Res..

[4] Saghafian F., Malmir H., Saneei P., Milajerdi A., Larijani B., Esmaillzadeh A. (2018). Fruit and vegetable consumption and risk of depression: Accumulative evidence from an updated systematic review and meta-analysis of epidemiological studies. Br. J. Nutr..

[5] Bilsen J. (2018). Suicide and Youth: Risk Factors. Front. Psychiatry.

[6] Viner R.M., Ross D., Hardy R., Kuh D., Power C., Johnson A., Wellings K., McCambridge J., Cole T.J., Kelly Y. (2015). Life course epidemiology: Recognising the importance of adolescence. J. Epidemiol. Community Health.

[7] Vos T., Lim S.S., Abbafati C., Abbas K.M., Abbasi M., Abbasifard M., Abbasi-Kangevari M., Abbastabar H., Abd-Allah F., Abdelalim A. (2020). Global burden of 369 diseases and injuries in 204 countries and territories, 1990–2019: A systematic analysis for the Global Burden of Disease Study 2019. Lancet.

[8] World Health Organizanization Mental Health and Substance Use. https://www.who.int/teams/mental-health-and-substance-use/suicide-data.

[9] Lopresti A.L., Hood S.D., Drummond P.D. (2013). A review of lifestyle factors that contribute to important pathways associated with major depression: Diet, sleep and exercise. J. Affect. Disord..

[10] Lassale C., Batty G.D., Baghdadli A., Jacka F.N., Sánchez-Villegas A., Kivimäki M., Akbaraly T.N. (2019). Healthy dietary indices and risk of depressive outcomes: A systematic review and meta-analysis of observational studies. Mol. Psychiatry.

[11] Altun A., Brown H., Szoeke C., Goodwill A.M. (2019). The Mediterranean dietary pattern and depression risk: A systematic review. Neurol. Psychiatry Brain Res..

[12] Lai J.S., Hiles S., Bisquera A., Hure A.J., McEvoy M., Attia J. (2013). A systematic review and meta-analysis of dietary patterns and depression in community-dwelling adults. Am. J. Clin. Nutr..

[13] Tarelho A., Duarte M., Melim J., Batista A., Almeida S. (2016). Dietary Pattern and Mental Health: Review of Literature. Eur. Psychiatry.

[14] Mason-D’Croz D., Bogard J.R., Sulser T.B., Cenacchi N., Dunston S., Herrero M., Wiebe K. (2019). Gaps between fruit and vegetable production, demand, and recommended consumption at global and national levels: An integrated modelling study. Lancet Planet. Health.

[15] Slavin J.L., Lloyd B. (2012). Health Benefits of Fruits and Vegetables. Adv. Nutr..

[16] Conner T.S., Brookie K.L., Carr A.C., Mainvil L.A., Vissers M.C.M. (2017). Let them eat fruit! The effect of fruit and vegetable consumption on psychological well-being in young adults: A randomized controlled trial. PLoS ONE.

[17] Saghafian F., Malmir H., Saneei P., Keshteli A.H., Hosseinzadeh-Attar M.J., Afshar H., Siassi F., Esmaillzadeh A., Adibi P. (2018). Consumption of fruit and vegetables in relation with psychological disorders in Iranian adults. Eur. J. Nutr..

[18] Ju S.-Y., Park Y.-K. (2019). Low fruit and vegetable intake is associated with depression among Korean adults in data from the 2014 Korea National Health and Nutrition Examination Survey. J. Health Popul. Nutr..

[19] Peltzer K., Pengpid S. (2017). Dietary consumption and happiness and depression among university students: A cross-national survey. J. Psychol. Afr..

[20] Wu S., Fisher-Hoch S.P., Reininger B.M., McCormick J.B. (2018). Association between fruit and vegetable intake and symptoms of mental health conditions in Mexican Americans. Health Psychol..

[21] Bhattacharyya M., Marston L., Walters K., D’Costa G., King M., Nazareth I. (2013). Psychological distress, gender and dietary factors in South Asians: A cross-sectional survey. Public Health Nutr..

[22] Kim T.-H., Choi J.-Y., Lee H.-H., Park Y. (2015). Associations between Dietary Pattern and Depression in Korean Adolescent Girls. J. Pediatr. Adolesc. Gynecol..

[23] Angelino D., Godos J., Ghelfi F., Tieri M., Titta L., Lafranconi A., Marventano S., Alonzo E., Gambera A., Sciacca S. (2019). Fruit and vegetable consumption and health outcomes: An umbrella review of observational studies. Int. J. Food Sci. Nutr..

[24] Głąbska D., Guzek D., Groele B., Gutkowska K. (2020). Fruit and Vegetable Intake and Mental Health in Adults: A Systematic Review. Nutrition.

[25] Goh C.M.J., Abdin E., Jeyagurunathan A., Shafie S., Sambasivam R., Zhang Y., Vaingankar J.A., Chong S.A., Subramaniam M. (2019). Exploring Singapore’s consumption of local fish, vegetables and fruits, meat and problematic alcohol use as risk factors of depression and subsyndromal depression in older adults. BMC Geriatr..

[26] Tsai A.C., Chang T.-L., Chi S.-H. (2011). Frequent consumption of vegetables predicts lower risk of depression in older Taiwanese-results of a prospective population-based study. Public Health Nutr..

[27] Payne M.E., Steck S.E., George R.R., Steffens D.C. (2012). Fruit, Vegetable, and Antioxidant Intakes Are Lower in Older Adults with Depression. J. Acad. Nutr. Diet..

[28] Głąbska D., Guzek D., Groele B., Gutkowska K. (2020). Fruit and vegetables intake in adolescents and mental health: A systematic review. Rocz. Państwowego Zakładu Hig..

[29] Khalid S., Williams C.M., Reynolds S. (2016). Is there an association between diet and depression in children and adolescents? A systematic review. Br. J. Nutr..

[30] McMartin S.E., Jacka F.N., Colman I. (2013). The association between fruit and vegetable consumption and mental health disorders: Evidence from five waves of a national survey of Canadians. Prev. Med..

[31] Palta P., Samuel L.J., Miller E.R., Szanton S.L. (2014). Depression and Oxidative Stress: Results from a meta-analysis of observational studies. Psychosom. Med..

[32] Maes M., De Vos N., Pioli R., Demedts P., Wauters A., Neels H., Christophe A. (2000). Lower serum vitamin E concentrations in major depression–Another marker of lowered antioxidant defenses in that illness. J. Affect. Disord..

[33] Miller A.L. (2008). The methylation, neurotransmitter, and antioxidant connections between folate and depression. Altern. Med. Rev..

[34] Jiménez-Fernández S., Gurpegui M., Díaz-Atienza F., Pérez-Costillas L., Gerstenberg M., Correll C.U. (2015). Oxidative Stress and Antioxidant Parameters in Patients with Major Depressive Disorder Compared to Healthy Controls Before and After Antidepressant Treatment: Results from a Meta-Analysis. J. Clin. Psychiatry.

[35] Nowak G. (2015). Zinc, future mono/adjunctive therapy for depression: Mechanisms of antidepressant action. Pharmacol. Rep..

[36] Fava M., Borus J.S., Alpert J.E., Nierenberg A.A., Rosenbaum J.F., Bottiglieri T. (1997). Folate, vitamin B_12_, and homocysteine in major depressive disorder. Am. J. Psychiatry.

[37] Ghadirian A.M., Ananth J., Engelsmann F. (1980). Folic acid deficiency and depression. J. Psychosom. Res..

[38] Reynolds E.H. (2002). Folic acid, ageing, depression, and dementia. BMJ.

[39] Rooney C., McKinley M., Woodside J. (2016). A systematic review of the potential role of fruit and vegetables in depression. Proc. Nutr. Soc..

[40] Rahe C., Unrath M., Berger K. (2014). Dietary patterns and the risk of depression in adults: A systematic review of observational studies. Eur. J. Nutr..

[41] Murakami K., Sasaki S. (2009). Dietary intake and depressive symptoms: A systematic review of observational studies. Mol. Nutr. Food Res..

[42] Sanhueza C., Ryan L., Foxcroft D. (2012). Diet and the risk of unipolar depression in adults: Systematic review of cohort studies. J. Hum. Nutr. Diet..

[43] Australian Bureau of Statistics Mental Health. https://www.abs.gov.au/statistics/health/health-conditions-and-risks/mental-health/latest-release.

[44] National Intitute of Mental Health Major Depression. https://www.nimh.nih.gov/health/statistics/major-depression.shtml.

[45] Frech A. (2012). Healthy behavior trajectories between adolescence and young adulthood. Adv. Life Course Res..

[46] Collins S., Dash S., Allender S., Jacka F., Hoare E. (2020). Diet and Mental Health During Emerging Adulthood: A Systematic Review. Emerg. Adulthood..

[47] Australian Bureau of Statistics National Survey of Mental Health and Wellbeing: Summary of Results. https://www.abs.gov.au/statistics/health/mental-health/national-survey-mental-health-and-wellbeing-summary-results/latest-release#articles.

[48] Liberati A., Altman D.G., Tetzlaff J., Murlow C., Gøtzsche P.C., Ioannidis J.P.A., Clarke M., Devereaux P.J., Kleijnen J., Moher D. (2009). The PRISMA Statement for Reporting Systematic Reviews and Meta-Analyses of Studies That Evaluate Health Care Interventions: Explanation and Elaboration. PLoS Med..

[49] Wells G.A., Shea B., O’Connell D., Peterson J., Welch V., Losos M., Tugwell P. The Newcastle-Ottawa Scale (NOS) for Assessing the Quality of Nonrandomised Studies in Meta-Analyses. http://www.ohri.ca/programs/clinical_epidemiology/oxford.asp.

[50] Lo C.K.-L., Mertz D., Loeb M. (2014). Newcastle-Ottawa Scale: Comparing reviewers’ to authors’ assessments. BMC Med. Res. Methodol..

[51] Choda N., Wakai K., Naito M., Imaeda N., Goto C., Maruyama K., Kadomatsu Y., Tsukamoto M., Sasakabe T., Kubo Y. (2020). Associations between diet and mental health using the 12-item General Health Questionnaire: Cross-sectional and prospective analyses from the Japan Multi-Institutional Collaborative Cohort Study. Nutr. J..

[52] Mujcic R., Oswald A.J. (2019). Does eating fruit and vegetables also reduce the longitudinal risk of depression and anxiety? A commentary on ’Lettuce be happy’. Soc. Sci. Med..

[53] Ocean N., Howley P., Ensor J. (2019). Lettuce be happy: A longitudinal UK study on the relationship between fruit and vegetable consumption and well-being. Soc. Sci. Med..

[54] Winzer R., Sorjonen K., Lindberg L. (2018). What Predicts Stable Mental Health in the 18–29 Age Group Compared to Older Age Groups? Results from the Stockholm Public Health Cohort 2002–2014. Int. J. Environ. Res. Public Health.

[55] Winpenny E.M., Van Harmelen A.-L., White M., Van Sluijs E.M., Goodyer I.M. (2018). Diet quality and depressive symptoms in adolescence: No cross-sectional or prospective associations following adjustment for covariates. Public Health Nutr..

[56] Hoare E., Hockey M., Ruusunen A., Jacka F.N. (2018). Does Fruit and Vegetable Consumption During Adolescence Predict Adult Depression? A Longitudinal Study of US Adolescents. Front. Psychiatry.

[57] Collin C., Assmann K.E., Andreeva V.A., Lemogne C., Hercberg S., Galan P., Kesse-Guyot E. (2016). Adherence to dietary guidelines as a protective factor against chronic or recurrent depressive symptoms in the French SU.VI.MAX cohort. Prev. Med..

[58] Kingsbury M., Dupuis G., Jacka F., Roy-Gagnon M.-H., McMartin S.E., Colman I. (2015). Associations between fruit and vegetable consumption and depressive symptoms: Evidence from a national Canadian longitudinal survey. J. Epidemiol. Community Health.

[59] Chang S.-C., Cassidy A., Willett W.C., Rimm E.B., O’Reilly E.J., Okereke O.I. (2016). Dietary flavonoid intake and risk of incident depression in midlife and older women. Am. J. Clin. Nutr..

[60] Akbaraly T.N., Sabia S., Shipley M.J., Batty G.D., Kivimäki M. (2013). Adherence to healthy dietary guidelines and future depressive symptoms: Evidence for sex differentials in the Whitehall II study. Am. J. Clin. Nutr..

[61] Chai W., Nigg C.R., Pagano I., Motl R.W., Horwath C.C., Dishman R.K. (2010). Associations of quality of life with physical activity, fruit and vegetable consumption, and physical inactivity in a free living, multiethnic population in Hawaii: A longitudinal study. Int. J. Behav. Nutr. Phys. Act..

[62] Sánchez-Villegas A., Delgado-Rodríguez M., Alonso A., Schlatter J., Lahortiga F., Majem L.S., Martínez-González M.A. (2009). Association of the Mediterranean Dietary Pattern With the Incidence of Depression: The Seguimiento Universidad de Navarra/University of Navarra follow-up (SUN) cohort. Arch. Gen. Psychiatry.

[63] Mujcic R., Oswald A.J. (2016). Evolution of Well-Being and Happiness After Increases in Consumption of Fruit and Vegetables. Am. J. Public Health.

[64] Seguí-Gómez M., De La Fuente C., Vázquez Z., De Irala J., A Martínez-González M. (2006). Cohort profile: The ‘Seguimiento Universidad de Navarra’ (SUN) study. Int. J. Epidemiol..

[65] Mihrshahi S., Dobson A.J., Mishra G.D. (2015). Fruit and vegetable consumption and prevalence and incidence of depressive symptoms in mid-age women: Results from the Australian longitudinal study on women’s health. Eur. J. Clin. Nutr..

[66] Emanuel A.S., McCully S.N., Gallagher K.M., Updegraff J.A. (2012). Theory of Planned Behavior explains gender difference in fruit and vegetable consumption. Appetite.

[67] De Wit L., Van Straten A., Van Herten M., Penninx B., Cuijpers P. (2009). Depression and body mass index, a u-shaped association. BMC Public Health.

[68] Révah-Levy A., Speranza M., Barry C., Hassler C., Gasquet I., Moro M.-R., Falissard B. (2011). Association between Body Mass Index and depression: The “fat and jolly” hypothesis for adolescents girls. BMC Public Health.

[69] Bountziouka V., Polychronopoulos E., Zeimbekis A., Papavenetiou E., Ladoukaki E., Papairakleous N., Gotsis E., Metal-linos G., Lionis C., Panagiotakos D. (2009). Long-term fish intake is associated with less severe depressive symptoms among elderly men and women: The MEDIS (MEDiterranean ISlands Elderly) epidemiological study. J. Aging Health.

[70] Timonen M., Horrobin D., Jokelainen J., Laitinen J., Herva A., Räsänen P. (2004). Fish consumption and depression: The Northern Finland 1966 birth cohort study. J. Affect. Disord..

[71] Li F., Liu X., Zhang D. (2015). Fish consumption and risk of depression: A meta-analysis. J. Epidemiol. Community Health.

[72] Offringa L.C., Stanton M.V., Hauser M.E., Gardner C.D. (2018). Fruits and Vegetables Versus Vegetables and Fruits: Rhyme and Reason for Word Order in Health Messages. Am. J. Lifestyle Med..

[73] Hebert J.R., Clemow L., Pbert L., Ockene I.S., Ockene J.K. (1995). Social Desirability Bias in Dietary Self-Report May Compromise the Validity of Dietary Intake Measures. Int. J. Epidemiol..

[74] Fried E.I. (2017). The 52 symptoms of major depression: Lack of content overlap among seven common depression scales. J. Affect. Disord..

[75] Radloff L.S. (1977). The CES-D Scale. A self-report depression scale for research in the general population. Appl. Psychol. Meas..

[76] Goldberg D.P., Blackwell B. (1970). Psychiatric Illness in General Practice: A Detailed Study Using a New Method of Case Identification. BMJ.

[77] Eaton W.W., Neufeld K., Chen L.-S., Cai G. (2000). A Comparison of Self-report and Clinical Diagnostic Interviews for Depression: Diagnostic interview schedule and schedules for clinical assessment in neuropsychiatry in the Baltimore epidemiologic catchment area follow-up. Arch. Gen. Psychiatry.

[78] Polaino A., Senra C. (1991). Measurement of depression: Comparison between self-reports and clinical assessments of depressed outpatients. J. Psychopathol. Behav. Assess..

[79] Uher R., Perlis R.H., Placentino A., Dernovšek M.Z., Henigsberg N., Mors O., Maier W., McGuffin P., Farmer A. (2012). Self-Report And Clinician-Rated Measures Of Depression Severity: Can One Replace The Other?. Depress. Anxiety.

[80] Quirk S.E., Williams L.J., O’Neil A., Pasco J.A., Jacka F.N., Housden S., Berk M., Brennan S.L. (2013). The association between diet quality, dietary patterns and depression in adults: A systematic review. BMC Psychiatry.

[81] Francis H., Stevenson R.J., Chambers J.R., Gupta D., Newey B., Lim C.K. (2019). A brief diet intervention can reduce symptoms of depression in young adults–A randomised controlled trial. PLoS ONE.

[82] Jacka F.N., O’Neil A., Opie R., Itsiopoulos C., Cotton S., Mohebbi M., Castle D., Dash S., Mihalopoulos C., Chatterton M.L. (2017). A randomised controlled trial of dietary improvement for adults with major depression (the ‘SMILES’ trial). BMC Med..

[83] Estaquio C., Kesse-Guyot E., Deschamps V., Bertrais S., Dauchet L., Galan P., Hercberg S., Castetbon K. (2009). Adherence to the French Programme National Nutrition Santé Guideline Score Is Associated with Better Nutrient Intake and Nutritional Status. J. Am. Diet. Assoc..

