# Supplementary material for: Association between Fruit and Vegetable Consumption and Depression Symptoms in Young People and Adults Aged 15–45: A Systematic Review of Cohort Studies
Source: Int J Environ Res Public Health. 2021 Jan 18;18(2):780. doi: 10.3390/ijerph18020780 (PMC7831325; doi:10.3390/ijerph18020780)
Supplement: Supplementary file 1 [file ijerph-18-00780-s001.pdf]

International Journal of
*Environmental Research and Public Health*

MDPI

**Table S1.** Inclusion and exclusion criteria for study designs.

| | Inclusion | Exclusion |
|---|---|---|
| **Population** | People 15-45 years of age<br>Healthy population | Presence of chronic disease including depression<br>Adults with nutritional needs different from the general population<br>Unique populations which are less likely to be representative of the general population (e.g., monks)<br>Animal studies |
| **Study Design** | Original cohort studies with a minimum follow up time of 12 months or longer | Cross sectional studies<br>Randomised control trials<br>Case-control studies<br>Reviews<br>Reports<br>Non-empirical research |
| **Time Frame** | 1 January 2000 to 31 August 2020 | Prior to 2000 |
| **Exposure** | Fruit and vegetable consumption, both independently and/or together | Fruit and vegetables were not analysed separately from other dietary patterns |
| **Outcome** | Depressive symptoms or Depression measured using validated questions, QoL, doctors diagnosis or medication use for depression | No valid measure of depressive symptoms/depression |

**Table S2.** Full electronic search strategy applied for Medline, Embase, and PsycInfo.

| Database | The applied full electronic search strategy |
|---|---|
| Medline via Ovid | 1. Young Adult/ |
| | 2. Adolescent/ or Adult/ |
| | 3. (young people or young adult* or early adult* or emerging adult* or adult*).ti,ab. |
| | 4. 1 or 2 or 3 |
| | 5. exp Fruit/ or "Fruit and Vegetable Juices"/ |
| | 6. Malus/ or Citrus sinensis/ or Pyrus/ or Vitis/ or Musa/ or exp Citrus/ or Rubus/ or Psidium/ or Actinidia/ |
| | 7. exp Vaccinium/ or Carica/ or cucurbitaceae/ or citrullus/ or Ananas/ or Fragaria/ or Carotenoids/ or exp Prunus/ |
| | 8. Rheum/ or Bombacaceae/ or Cocos/ or Citrullus/ |
| | 9. (fruit* or apple* or orange* or pear or pears or grape or grapes or banana* or berry or berries or citrus).ti,ab. |
| | 10. (blackberr* or cranberr* or guava* or kiwi* or lingonberr* or papaya* or mandarin* or tangerine* or melon* or pineapple*).ti,ab. |
| | 11. (raspberr* or strawberr* or grapefruit* or satsuma* or plum or plums or apricot* or peach or peaches or rhubarb or durian or cherry or cherries).ti,ab. |
| | 12. (nectarine* or coconut* or dragonfruit* or watermelon*).ti,ab. |
| | 13. (fruit adj1 (intake* or consumption*)).ti,ab. |
| | 14. 5 or 6 or 7 or 8 or 9 or 10 or 11 or 12 or 13 |
| | 15. Vegetables/ or vegetarians/ or vegans/ or Plant Leaves/ |
| | 16. Daucus carota/ or Lycopersicon esculentum/ or Solanum tuberosum/ or exp allium/ or onions/ |
| | 17. Apium/ or Spinacia oleracea/ or peas/ or soybeans/ or Phaseolus/ or Fabaceae/ or exp Chenopodiaceae/ or exp Brassica/ |
| | 18. Cucumis sativus/ or Salads/ or Solanum melongena/ or Capsicum/ or Abelmoschus/ or Cucurbita/ |
| | 19. Cynara scolymus/ or Lettuce/ or Pastinaca/ or Asparagus Plant/ or Cicer/ or Foeniculum/ or seaweed/ or kelp/ |
| | 20. Ipomoea batatas/ or Agaricales/ or Nuts/ or Soy Foods/ or "Fermented Foods and Beverages"/ |
| | 21. (vegetable* or vegan* or vegetarian* or leafy green* or carrot* or cabbage* or brassica* or tomato or tomatoes or potato or potatoes).ti,ab. |
| | 22. (onion* or celery or spinach or pea or peas or bean or beans or broccoli or cauliflower* or beetroot* or turnip* or cucumber).ti,ab. |

| Database | The applied full electronic search strategy |
|---|---|
| Medline via Ovid | 24. (kale or parsnip* or asparagus or chickpea* or fennel or seaweed* or sweet potato* or kimchi or mushroom* or tofu or tempeh).ti,ab. |
| | 25. (vegetable adj1 (intake* or consumption*)).ti,ab. |
| | 26. 15 or 16 or 17 or 18 or 19 or 20 or 21 or 22 or 23 or 24 or 25 |
| | 27. diet/ or diet, mediterranean/ or diet, vegetarian/ or diet, vegan/ or diet, western/ or diet, healthy/ |
| | 28. Dietary Fiber/ or Antioxidants/ or Nutritional Status/ or Folic Acid/ or Micronutrients/ or Nutrition Surveys/ or Nutrition Assessment/ or "diet, food, and |

| | |
|---|---|
| | nutrition"/ |
| | 29. (diet* or healthy diet or nutrition* or juice*).ti,ab. |
| | 30. ((fruit and vegetable) adj1 (intake or consumption)).ti,ab. |
| | 31. 27 or 28 or 29 or 30 |
| | 32. 14 or 26 or 31 |
| | 33. affective symptoms/ or depression/ or mental fatigue/ or mental disorders/ or anxiety disorders/ or mood disorders/ |
| | 34. Depressive Disorder, Major/ or Depressive Disorder/ or Dysthymic Disorder/ or Seasonal Affective Disorder/ |
| | 35. Panic Disorder/ or Panic/ or self mutilation/ or exp suicide/ or exp stress, psychological/ or fear/ or frustration/ or loneliness/ or sadness/ |
| | 36. (depression or depressive or anxiety or anxious or psychological distress or worries or sad or unhappy or unhappiness or helpless* or hopeless*).ti,ab. |
| | 37. (suicide* adj2 (thought* or idea* or think* or attempt*)).ti,ab. |
| | 38. (self adj1 (injury or harm or hurt)).ti,ab. |
| | 39. healthy lifestyle/ or Life Change Events/ or Mental Health/ or affect/ or irritable mood/ or anxiety/ |
| | 40. (automutilation or alienation or lonely or depressed or well-being or well being).ti,ab. |
| | 41. 33 or 34 or 35 or 36 or 37 or 38 or 39 or 40 |
| | 42. epidemiologic studies/ or exp cohort studies/ |
| | 43. (cohort stud* or cohort analysis).ti,ab. |
| | 44. (follow up adj1 (study or studies)).ti,ab. |
| | 45. (longitudinal adj1 (study or studies)).ti,ab. |
| | 46. 42 or 43 or 44 or 45 |
| | 47. 4 and 32 and 41 and 46 |
| | 48. limit 47 to (english language and humans and yr="2000 - 2020") |
| **Database** | **The applied full electronic search strategy** |
| Embase via Ovid | 1. young adult/ or adult/ or adolescent/ |
| | 2. (young people or young adult* or early adult* or emerging adult* or adult*).ti,ab. |
| | 3. adolescence/ or adulthood/ |
| | 4. 1 or 2 or 3 |
| | 5. fruit juice/ or fruit consumption/ or fruit vegetable/ or exp fruit/ |
| | 6. apple juice/ or apple/ or sweet orange/ or orange juice/ |
| | 7. durian fruit/ or passion fruit/ or "lime (fruit)"/ or "orange (fruit)"/ or Evodia fruit/ or "date (fruit)"/ or pome fruit/ or citrus fruit/ or miracle fruit/8. pear juice/ or pear/ or grape juice/ or grape/ or banana/ or berry juice/ or berry/ |
| | 9. citrus/ or grapefruit/ or lemon/ or mandarin/ or satsuma/ or tangerine/ or guava/ or blackberry/ or cranberry juice/ or cranberry/ or guava/ |
| | 10. avocado/ or berry/ or black currant/ or blueberry/ or breadfruit/ or gooseberry/ or huckleberry/ or kiwifruit/ or lingonberry/ or loquat/ or lychee/ or mango/ or |

| | |
|---|---|
| | miracle fruit/ or muskmelon/ or papaya/ or passion fruit/ or persimmon/ or pineapple/ or pome fruit/ or pomegranate/ or raspberry/ or strawberry/ or tomato/ or watermelon/ |
| | 11. mango juice/ or lime juice/ or beetroot juice/ or watermelon juice/ or plant juice/ or lemon juice/ or apple juice/ or cherry juice/ or aloe juice/ or mandarin juice/ or tomato juice/ or pineapple juice/ |
| | 12. strawberry juice/ or grapefruit/ or grapefruit juice/ or plum/ or apricot/ or peach/ or rhubarb/ or nectarine/ or coconut/ or Hylocereus undatus/ |
| | 13. (fruit* or apple* or orange* or pear or pears or grape or grapes or banana* or berry or berries or citrus).ti,ab. |
| | 14. (blackberr* or cranberr* or guava* or kiwi* or lingonberr* or papaya* or mandarin* or tangerine* or melon* or pineapple*).ti,ab. |
| | 15. (raspberr* or strawberr* or grapefruit* or satsuma* or plum or plums or apricot* or peach or peaches or rhubarb or durian or cherry or cherries).ti,ab. |
| | 16. (nectarine* or coconut* or dragonfruit* or watermelon*).ti,ab. |
| | 17. (fruit adj1 (intake* or consumption*)).ti,ab. |
| | 18. 5 or 6 or 7 or 8 or 9 or 10 or 11 or 12 or 13 or 14 or 15 or 16 or 17 |
| | 19. vegetable/ or "root vegetable"/ or pickled vegetable/ or leafy vegetable/ or stem vegetable/ or vegetable consumption/ or vegetable juice/ |
| | 20. vegan diet/ or vegan/ or vegetarian diet/ or vegetarian/ or fermented soybean/ or tofu/ or kimchi/ |
| | 21. carrot juice/ or carrot/ or Chinese cabbage/ or celery cabbage/ or cabbage/ or white cabbage/ or red cabbage/ or Brassica/ or potato/ or sweet potato/ or onion/ or celery/ |

| **Database** | **The applied full electronic search strategy** |
|---|---|
| **Embase via Ovid** | 22. spinach/ or pea/ or bean/ or legume/ or soybean/ or broccoli/ or cauliflower/ or beetroot juice/ or beetroot/ or turnip/ or cucumber/ or salad/ or leek/ or aubergine/ |
| | 23. Romaine lettuce/ or lettuce/ or leaf lettuce/ or iceberg lettuce/ or kale/ or Chinese kale/ or parsnip/ or asparagus/ or chickpea/ or fennel/ |
| | 24. seaweed/ or edible mushroom/ or pepper/ or okra/ or squash/ or artichoke/ |
| | 25. (vegetable* or vegan* or vegetarian* or leafy green* or carrot* or cabbage* or brassica* or tomato or tomatoes or potato or potatoes).ti,ab. |
| | 26. (onion* or celery or spinach or pea or peas or bean or beans or broccoli or cauliflower* or beetroot* or turnip* or cucumber).ti,ab. |
| | 27. (salad or salads or legume* or leek or leeks or aubergine* or pepper* or okra or pumpkin* or squash* or artichoke* or lettuce*).ti,ab. |
| | 28. (kale or parsnip* or asparagus or chickpea* or fennel or seaweed* or sweet potato* or kimchi or mushroom* or tofu or tempeh).ti,ab. |
| | 29. (vegetable adj1 (intake* or consumption*)).ti,ab. |
| | 30. 19 or 20 or 21 or 22 or 23 or 24 or 25 or 26 or 27 or 28 or 29 |
| | 31. diet/ or high fiber diet/ or mediterranean diet/ or western diet/ |
| | 32. exp healthy diet/ or nutrient/ or nutrient intake/ or trace element/ or antioxidant/ or folic acid/ or dietary fiber/ or fruit vegetable/ |
| | 33. nutrition/ or adolescent nutrition/ or dietary intake/ or dietary pattern/ or food intake/ or nutritional assessment/ |
| | 34. (fruit and vegetable).ti,ab. |
| | 35. (diet* or healthy diet or nutrition* or juice*).ti,ab. |
| | 36. ((fruit and vegetable) adj1 (intake or consumption)).ti,ab |
| | 37. 31 or 32 or 33 or 34 or 35 or 36 |

| | |
|---|---|
| | 38. 18 or 30 or 37 |
| | 39. depression inventory/ or minor depression/ or Self-rating Depression Scale/ or "mixed anxiety and depression"/ or major depression/ or depression/ or adolescent depression/ or depression assessment/ or mental health/ |
| | 40. depression/ or seasonal affective disorder/ or dysthymia/ or panic/ or mental disease/ or anxiety disorder/ or emotional disorder/ or mood disorder/ |
| | 41. suicidal behavior/ or suicidal ideation/ or suicide/ or suicide attempt/ or automutilation/ or frustration/ or loneliness/ or sadness/ |
| **Database** | **The applied full electronic search strategy** |
| **Embase via Ovid** | 42. emotion/ or affect/ or anger/ or fear/ or helplessness/ or hopelessness/ or mood/ or mood change/ or nervousness/ or unhappiness/<br>43. (depression or depressive or anxiety or anxious or psychological distress or worries or sad or unhappy or unhappiness or helpless* or hopeless*).ti,ab.<br>44. (symptom* of depression or depressive symptom*).ti,ab.<br>45. (suicide* adj2 (thought* or idea* or think* or attempt*)).ti,ab.<br>46. (self adj1 (injury or harm or hurt)).ti,ab.<br>47. healthy lifestyle/<br>48. life event/<br>49. ("loss of interest" or alienation or lonely or depressed or well-being or well being).ti,ab.<br>50. 39 or 40 or 41 or 42 or 43 or 44 or 45 or 46 or 47 or 48 or 49<br>51. epidemiology/ or cohort analysis/ or longitudinal study/ or follow up/<br>52. (cohort stud* or cohort analysis).ti,ab.<br>53. (follow up adj1 (study or studies)).ti,ab.<br>54. (longitudinal adj1 (study or studies)).ti,ab.<br>55. 51 or 52 or 53 or 54<br>56. 4 and 38 and 50 and 55<br>57. limit 56 to (human and english language and yr="2000 -2020") |
| PsycInfo via Ovid | 1. Adult Offspring/<br>2. (young people or young adult* or early adult* or emerging adult* or adult*).ti,ab.<br>3. Emerging Adulthood/<br>4. 1 or 2 or 3<br>5. Diets/ or Food Intake/ or Food Preferences/ or Nutrition/<br>6. (fruit* or apple* or orange* or pear or pears or grape or grapes or banana* or berry or berries or citrus).ti,ab.<br>7. (blackberr* or cranberr* or guava* or kiwi* or lingonberr* or papaya* or mandarin* or tangerine* or melon* or pineapple*).ti,ab.<br>8. (raspberr* or strawberr* or grapefruit* or satsuma* or plum or plums or apricot* or peach or peaches or rhubarb or durian or cherry or cherries).ti,ab.<br>9. (nectarine* or coconut* or dragonfruit* or watermelon*).ti,ab. |

| Database | The applied full electronic search strategy |
|---|---|
| PsycInfo via Ovid | 10. (fruit adj1 (juice* or consumption or intake)).ti,ab.<br>11. (vegetable* or vegan* or vegetarian* or leafy green* or carrot* or cabbage* or brassica* or tomato or tomatoes or potato or potatoes).ti,ab.<br>12. (onion* or celery or spinach or pea or peas or bean or beans or broccoli or cauliflower* or beetroot* or turnip* or cucumber).ti,ab.<br>13. (salad or salads or legume* or leek or leeks or aubergine* or pepper* or okra or pumpkin* or squash* or artichoke* or lettuce*).ti,ab.<br>14. (kale or parsnip* or asparagus or chickpea* or fennel or seaweed* or sweet potato* or kimchi or mushroom* or tofu or tempeh).ti,ab.<br>15. "fruit and vegetable".ti,ab.<br>16. (vegetable adj1 (juice* or consumption or intake)).ti,ab.<br>17. Folic Acid/<br>18. Antioxidants/<br>19. (nutritional adj1 (assessment* or survey*)).ti,ab.<br>20. (mediterranean diet or healthy diet or dietary pattern* or dietary intake).ti,ab.<br>21. ((fruit and vegetable) adj1 (intake or consumption)).ti,ab.<br>22. 5 or 6 or 7 or 8 or 9 or 10 or 11 or 12 or 13 or 14 or 15 or 16 or 17 or 18 or 19 or 20 or 21<br>23. major depression/ or affective disorders/ or dysthymic disorder/ or seasonal affective disorder/<br>24. Adolescent Development/ or Anxiety Disorders/ or "Depression (Emotion)"/<br>25. mental health/ or mental status/ or "mental health and illness assessment"/ or well being/<br>26. mental disorders/ or psychological assessment/ or suicide/<br>27. negative emotions/ or fatigue/ or Life Changes/ or Stress/ or psychological stress/<br>28. emotional states/ or alienation/ or anxiety/ or distress/ or fear/ or frustration/ or hopelessness/ or loneliness/ or pessimism/ or restlessness/ or sadness/ or suffering/<br>29. (depression or depressive or anxiety or anxious or psychological distress or worries or sad or unhappy or unhappiness or helpless* or hopeless*).ti,ab.<br>30. ("symptom* of depression" or "depressive symptom*").ti,ab.<br>31. (suicide* adj2 (thought* or idea* or think* or attempt*)).ti,ab.<br>32. (self adj1 (injury or harm or hurt)).ti,ab.<br>33. ("loss of interest" or alienation or lonely or depressed or well-being or well being).ti,ab. |
| **Database** | **The applied full electronic search strategy** |
| **PsycInfo via Ovid** | 34. 23 or 24 or 25 or 26 or 27 or 28 or 29 or 30 or 31 or 32 or 33<br>35. cohort analysis/ or longitudinal studies/ or prospective studies/ or followup studies/ or epidemiology/<br>36. (cohort stud* or cohort analysis).ti,ab.<br>37. (follow up adj1 (study or studies)).ti,ab.<br>38. (longitudinal adj1 (study or studies)).ti,ab.<br>39. 35 or 36 or 37 or 38 |

| |
|---|
| 40. 4 and 22 and 34 and 39 |
| 41. limit 40 to (english language and yr="2000 -2020") |

**Table S3.** Justification of exclusion at full text screening

| Reason for exclusion | Number of studies | Excluded studies reference | | | |
|---|---|---|---|---|---|
| Incorrect study design:<br>Cross sectional<br>Case-control<br>Randomised control trial<br>Poster format<br>Abstract only available | n= 62 | Godos J, et al., 2019<br>Emerson SD, et al., 2019<br>Schultchen D, et al., 2019<br>Abildsnes E, et al. 2017<br>Adjibade M, et al. 2017<br>Herbison CE, et al. 2012<br>Szabo de Edelenyi F, et al., 2020<br>Moreno-Agostino D, et al., 2019<br>Sánchez-Villegas A, et al. 2009<br>Esteban-Gonzalo L, et al., 2019<br>Sangsefidi ZS, Mirzaei M, et al., 2020<br>Matta J, Hoertel N, et al., 2020<br>Gascoyne CR, et al., 2019<br>Gascoyne CR, Simpson Jr S, et al., 2019 | Saghafian F, et al., 2019<br>Paans NPG, et al., 2019<br>Moludi J, et al., 2020<br>Matta J, et al., 2018<br>Solmi M, et al., 2019<br>Paslakis G, et al., 2020<br>Miyake Y, et al., 2018<br>Hajianfar H, et al., 2020<br>Alfawas W, et al., 2019<br>Bryan J,et al. 2004<br>Richard A, et al. 2015<br>Won MS,et al. 2016<br>Ackard DM, et al. 2002<br>Konttinen H, et al. 2010<br>Gregorio MJ, et al. 2017<br>Wesselman LMP, et al., 2019 | Matta J, et al., 2019<br>Pfeiler TM, et al., 2020<br>Sousa KT, et al., 2019<br>Philips A, et al., 2020<br>Lee J, et al., 2020<br>Wu H, et al., 2020<br>Ju SY, et al., 2019<br>Sangsefidi ZS, et al., 2020<br>Sakai H, et al. 2017<br>Miki T, et al. 2015<br>Beydoun MA, et al 2010<br>Watanabe H, et al 2012<br>Ahmed N, et al. 2017<br>Beydoun MA, et al. 2010<br>Yary T. et al. 2013<br>Bergmans RS, et al. 2017 | Liu C, et al. 2007<br>Watanabe H, et al. 2011<br>Adams TB, et al. 2008<br>Shakya PR, et al., 2020<br>Grases G, et al., 2019<br>Avalos LA, et al., 2020<br>Ashurst J, et al., 2018<br>Liao Y, et al., 2019<br>Shin Y, et al., 2019<br>Wu S, et al., 2018<br>Patel S, et al., 2019<br>Nanri A, et al. 2010<br>Fabian C, et al. 2013<br>Bertram L, et al. 2014<br>Wirth MD, et al. 2017<br>Wirth M, et al. 2016 |
| Incorrect population<br>Started with depression<br>Had existing medical issues<br>Age only >45 years old<br>Age only < 14 years old<br>Most participants in population is older people<br>Unique population | n= 26 | Ramin S, et al., 2020<br>Nathanson R, et al., 2018<br>Pengpid S, et al., 2019<br>Elstgeest LEM, et al., 2019<br>Cherian L, et al., 2020<br>Sanchez-Villegas A, et al. 2006<br>Sanchez-Villegas A, et al. 2017 | Youri J et al 2016<br>Notara V, et al. 2016<br>Tolmunen T, et al. 2004<br>Walsh JL, et al. 2013<br>Hingle MD, et al. 2014<br>Adjibade M, et al. 2017<br>Baek D, et al. 2013 | Huang P, et al., 2019<br>Loewen OK, et al., 2019<br>Boehm JK, et al., 2018<br>Kobayashi LC, et al., 2018<br>Lavalee K, et al., 2019<br>Matthews KA, et al., 2019 | Nanri A, et al. 2013<br>Ruusunen A, et al. 2014<br>Ribeiro SML, et al. 2017<br>Lai JS, et al. 2017<br>Cheng HY, et al., 2019<br>Hoare E et al., 2020 |

| Reason for exclusion | Number of studies | Excluded studies reference | | | |
|---|---|---|---|---|---|
| Incorrect intervention/exposure<br>Fruit and vegetables not analysed separate from diet<br>Only looking at specific nutrients | n= 36 | Adjibade M, et al., 2019<br>Adjibabe M, Lemogne C, et al., 2019<br>Adjibabe M, Lemogne C, Julia C, et al., 2019<br>Sanchez-Villegas A, Ruiz-Canela M, et al. 2015<br>Sharpe PA, et al. 2016<br>Northstone K, et al. 2018<br>Smith-Marek EN et al. 2016<br>Vermeulen E, et al. 2016<br>Shakya PR, et al., 2020 | Opie RS, et al., 2020<br>Recchia D, et al., 2020<br>Nanri A, et al., 2020<br>Marozoff S, et al., 2020<br>Oftedal S, et al., 2020<br>Schweren LJS, et al., 2020<br>Oddy WH, et al., 2018<br>Carlos S, et al., 2018<br>Northstone K, et al., 2020<br>Guo F, et al., 2019<br>Sanchez-Villegas A et al. 2015 | Fresan U, et al., 2019<br>Wilson CA, et al., 2020<br>Velten J, et al., 2018<br>Ruiz-Estigarribia L, et al., 2019<br>Miki T, et al., 2018<br>Derom ML, et al. 2012<br>Kesse-Guyot E, et al. 2013<br>Nanri A, et al. 2013<br>Pisinger C, et al. 2016 | Henriquez Sanchez P 2012<br>Beydoun MA, et al. 2016<br>Kurspahic-Mujcic A, et al. 2014<br>Astorg P, et al. 2008<br>Gall SL, et al. 2016<br>Mouchacca J, et al. 2013<br>Akbaraly T, et al. 2016 |
| Incorrect outcome<br>Looked at key life transitions<br>Looked at cognitive functioning<br>Looked at happiness<br>Looked at emotion | n= 8 | Winpenny EM, et al., 2018<br>Kesse-Guyot E, et al. 2014 | Mujcic R, et al. 2016<br>Haibach JP, et al 2016 | Allen MS, et al. 2016<br>Kim O, et al. 2017 | Haibach JP, et al. 2015<br>Ashurst J, et al. 2016 |

**Table S4.** The Newcastle-Ottawa Scale results.

| Author | Selection* | | | | Comparability‡ | | Outcome† | | | Total§ |
|---|---|---|---|---|---|---|---|---|---|---|
| | Representativeness of the exposed cohort | Selection of the non-exposed cohort | Ascertainment of exposure | Demonstration that outcome of interest was not present at start of study | Adjust for the most important risk factors | Adjust for other risk factors | Assessment of outcome | Follow-up adequacy | Adequacy of follow up of cohorts | |
| **Choda et al., 2020** | 1 | 1 | 0 | 1 | 1 | 1 | 0 | 1 | 1 | 7 Good |
| Mujcic and Oswald, 2019 | 1 | 1 | 0 | 1 | 1 | 1 | 0 | 1 | 1 | 7 Good |
| Ocean et al., 2019 | 1 | 1 | 0 | 0 | 1 | 1 | 0 | 1 | 1 | 6 Good |
| Winzer et al., 2018 | 1 | 1 | 0 | 1 | 1 | 1 | 0 | 1 | 0 | 6 Good |
| Winpenny et al., 2018 | 0 | 1 | 0 | 1 | 1 | 1 | 0 | 1 | 1 | 6 Good |
| Hoare et al., 2018 | 1 | 1 | 0 | 1 | 1 | 1 | 0 | 1 | 0 | 6 Good |
| Chang et al., 2016 | 0 | 1 | 0 | 1 | 1 | 1 | 0 | 1 | 1 | 6 Good |
| Collin et al., 2016 | 0 | 1 | 0 | 1 | 1 | 1 | 0 | 1 | 0 | 5 Moderate |
| Kingsbury et al., 2016 | 1 | 1 | 1 | 1 | 1 | 1 | 0 | 1 | 1 | 8 Very Good |
| Akbaraly et al., 2013 | 0 | 1 | 0 | 1 | 1 | 1 | 0 | 1 | 1 | 6 Good |
| Chai et al., 2010 | 1 | 0 | 0 | 0 | 1 | 1 | 0 | 1 | 0 | 4 Moderate |
| Sanchez-Villegas et al., 2009 | 0 | 1 | 0 | 1 | 1 | 1 | 0 | 1 | 1 | 6 Good |

*Maximum 4 points awarded for cohort representativeness, selection of non-exposed cohort, exposure assessment and demonstration outcome not present at baseline.
‡Maximum 2 points awarding for controlling for the primary confounding variable and the secondary confounding variables.
†Maximum 3 points awarded for follow-up length, adequacy of follow-up and outcome assessment.
§A maximum of 9 points could be awarded.
Category:
Very Good quality = 8-9 points; Good quality = 6-7 points; Moderate quality = 4-5 points; Low quality = 0-3 points
